# Perspectives on the Role of Endocannabinoids in Autism Spectrum Disorders

**DOI:** 10.21926/obm.neurobiol.1702005

**Published:** 2017-04-27

**Authors:** Mason L. Yeh, Eric S. Levine

**Affiliations:** Department of Neuroscience, University of Connecticut School of Medicine, 263 Farmington Avenue, Farmington, CT 06032, USA

**Keywords:** Endocannabinoids, Autism, Development, Cortex

## Abstract

Autism spectrum disorders (ASDs) are diagnosed on the basis of three behavioral features, namely, (1) deficits in social communication, (2) absence or delay in language and (3) stereotypy. The consensus regarding the neurological pathogenesis of ASDs is aberrant synaptogenesis and synapse function. Further, it is now widely accepted that ASD is neurodevelopmental in nature, placing emphasis on derangements occurring at the level of intra- and intercellular signaling during corticogenesis. At present, there is an ever-growing list of mutations in putative susceptibility genes in affected individuals, preventing effective transformation of knowledge gathered from basic science research to the clinic. In response, the focus of ASD biology has shifted toward the identification of cellular signaling pathways that are common to various ASD-related mutations in hopes that these shared pathways may serve as more promising treatment targets than targeting individual genes or proteins. To this end, the endogenous cannabinoid (endocannabinoid, eCB) system has recently emerged as a promising therapeutic target in the field of ASD research. The eCB system is altered in several neurological disorders, but the role of these bioactive lipids in ASD etiology remains poorly understood. In this perspective, we review current evidence linking eCB signaling to ASDs and put forth the notion that continued focus on eCBs in autism research may provide valuable insight into pathophysiology and treatment strategies. In addition to its role in modulating transmitter release at mature synapses, the eCB signaling system plays important roles in many aspects of cortical development, and disruption of these effects of eCBs may also be related to ASD pathophysiology.

## Introduction

Autism spectrum disorders (ASDs) comprise a continuum of symptoms and levels of disability that exhibit an early onset and persist through adulthood. ASDs are characterized by deficits in communication and social interaction, as well as by stereotypic behaviors, restricted patterns of interest and sensory issues. Two features distinguish ASDs from other behavioral disorders, clinical and pathogenetic heterogeneity and the distribution of autistic features as a continuum in the general population, dissolving the distinction between “affected” and “unaffected” [1,2]. There are currently no behavioral, genetic, imaging or electrophysiological assays that can specifically diagnose ASDs in the clinic. The prevailing hypothesis in central nervous system (CNS) research is that ASDs may be due to a disruption of the normal process of experience-dependent synaptic development. Several challenges arise, however, in translating our current knowledge base to the clinic. ASD-causing mutations in individual genes that have been uncovered are extremely rare, where each accounts for ~1% of ASD patients. Genome analyses have also revealed the association of copy-number variants and single-nucleotide variants with ASDs. A number of these variants are *de novo* (not present in either parent). Whole-exome sequencing studies estimate between 400 to 1000 ASD susceptibility genes [3]. In addition to germline mutations, somatic mutations that affect a small subset of neurons can contribute to neurological disorders such as epilepsy, brain malformations and possibly ASDs [4]. In light of these difficulties in determining genetic markers of ASD, current research has moved toward examining cell signaling pathways that may be commonly affected across known ASD-related gene mutations. For example, the metabotropic glutamate receptor (mGluR) and mammalian target of rapamycin (mTOR) pathways have been targeted for developing therapeutic strategies in treating patients with ASDs [5]. Evidence from several mouse models of ASD-related disorders, including Fragile-X Syndrome (FXS) [6–9] and Tuberous Sclerosis (TSC) [10,11] suggest that dysregulation of mTOR and mGluR signaling pathways contribute to deficits in synaptic plasticity and learning and memory. Additionally, there are several converging lines of evidence that indicate mGluR signaling is downstream of mTOR signaling in regulating long-term potentiation (LTP) [12,13] and long-term depression (LTD) [10,11].

Similarly, recent studies provide compelling evidence that disruption of endogenous cannabinoid (endocannabinoid, eCB) signaling may be responsible for synaptic deficits that underlie cognitive and behavioral deficits associated with ASDs. The eCB system has been shown to control emotional responses [14], behavioral reactivity to context [15] and social interaction [16]. Therefore, it is plausible that alterations to eCB signaling may contribute to the autistic phenotype. In this perspective, we review current evidence linking eCB signaling to ASDs and put forth the notion that continued focus on eCBs in autism research may provide valuable insight into pathophysiology and treatment strategies. In addition to its role in modulating transmitter release at mature synapses, the eCB signaling system plays important roles in many aspects of cortical development, and disruption of these effects of eCBs may also be related to ASD pathophysiology. These observations span both rodent and human models *in vitro* and *in vivo*. Furthermore, the scope of studies range from eCB-dependent signaling mechanisms, such as synaptic plasticity, to behavioral studies examining social reward processing. However, the precise relationship between the eCB system and ASD pathophysiology remains to be clearly delineated.

## The Endocannabinoid System

The endogenous cannabinoid (eCB) system is comprised of cannabinoid receptors, endogenous ligands, and their corresponding synthetic and metabolic enzymes. The effects of eCBs are primarily mediated by binding cannabinoid type 1 (CB1) and type 2 (CB2) receptors. Both CB1 and CB2 receptors are G protein-coupled receptors, which couple mainly to G proteins of the G_i_ and G_o_ classes [17]. Activation of these subclasses of G proteins inhibits adenylyl cyclase and certain voltage-dependent calcium channels and activates inwardly-rectifying potassium channels regulated by mitogen-activated protein kinases. In conjunction, CB1 and CB2 receptor activation exerts diverse consequences across cellular physiology [17].

Neuroanatomical studies have reported abundant CB1 receptor expression in the developing telencephalon as early as embryonic day (E) 11.5 in mouse [18]. The strongest levels of CB1 receptor mRNA at this stage are present in pioneer neurons that occupy the marginal zone of the dorsal cortex, which include Cajal-Retzius cells that are best-known for their role as an instructive signaling cue through secretion of Reelin [19,20]. During mid-stages of cortical development (E13.5–E14.5) in mouse, CB1 receptor expression appears to be upregulated in the intermediate zone (IZ), while continuing to show robust expression in immature neurons accumulating in the overlying cortical plate (CP) [20,21]. Concomitantly, in the ventral forebrain, CB1 receptor expression has been reported in the subpial region of the ganglionic eminence (GE), which gives rise to cortical interneurons, and the primordium of the hippocampus [22]. At later stages of cortical development, CB1 receptor expression is heterogeneously distributed throughout cortical laminae and the hippocampus in both GABAergic and glutamatergic neurons [23–25].

The expression pattern of CB1 receptors has also been examined in human fetal brain using *in situ* hybridization and binding assays, which provide evidence of strong CB1 receptor expression in the developing cerebral cortex, hippocampus, caudate nucleus, putamen, and cerebellum [26]. Expression of the CB1 receptor has been reported as early as gestational week (GW) 9 in the subventricular zone (SVZ) of the lateral ventricle and Cajal-Retzius cells occupying the MZ, similar to reports in mouse. During the second trimester of gestation, dense labeling of CB1 receptor mRNA is present in fiber-enriched tracts, which, in the adult brain, are practically devoid of CB1 receptor expression [26]. This transient pattern of expression provides further support of the division between eCB signaling during cortical development and identifies the eCB system as a promising target for further examination in the context of neurodevelopmental disorders [27].

The first eCB ligands to be discovered and consequently best characterized are arachidonoyl ethanolamide (anandamide; AEA) and 2-arachidonoyl glycerol (2-AG). A notable feature of both anandamide and 2-AG is that their precursors are present in the lipid membrane. While both anandamide and 2-AG are derivatives of arachidonic acid, their routes of synthesis and degradation *in vivo* are entirely distinct and mediated by a separate set of enzymes [28]. Anandamide is produced from N-arachidonoyl phosphatidyl ethanol (NAPE) and studies have uncovered two routes of synthesis in the central nervous system: (1) NAPE-PLD [29]; and (2) NAPEphospholipase C (PLC) followed by phosphatase [30]. The synthetic pathway for 2-AG involves sequential hydrolysis of an arachidonoyl-containing phosphatidyl inositol bis-phosphate by PLCβ, followed by subsequent hydrolysis of the resulting diacylglycerol by diacylglycerol lipase (DAGL) [31]. Hydrolysis of an arachidonoyl-containing phosphatidyl inositol bis-phosphate by PLCβ is initiated following stimulation of receptors that activate PLC, such as type I metabotropic glutamate receptors (mGLuRs), M1 or M3 muscarinic receptors, or orexin A, which often leads to production of 2-AG. Two isoforms of DAGL have been identified in nervous tissue, DAGLα and DAGLβ, where DAGLα appears to be the isoform responsible for most 2-AG production that contributes to synaptic plasticity in the mature brain [32–34]. The breakdown of anandamide to AA and ethanolamine is due mainly to the serine hydrolase fatty acid amide hydrolase (FAAH) and FAAH-2 [35,36]. Degradation of 2-AG to AA and glycerol is mediated by monoacylglycerol lipase (MAGL) [37].

## CB1 Receptor Signaling in Cortical Development

The eCB system is involved in many aspects of early neuronal development and differentiation. Components of the eCB system (including receptors, ligands, synthesizing enzymes and degradative enzymes) are expressed in the developing brain prior to synaptic maturation and neuronal activity (reviewed in [38]). Here, we focus on summarizing CB1 receptor signaling during cortical development and downstream signaling mechanisms that have been shown to play a role in neural progenitor cell maintenance, differentiation and maturation, specifically pertaining to cortical glutamatergic pyramidal neurons. While CB2 receptors may also be involved in cortical development and modulating synaptic activity, their precise role in neuronal signaling remains to be clearly demonstrated [39].

CB1 receptor activity regulates neuronal progenitor cell proliferation and survival. *In vivo* studies have shown that CB1 receptor loss of function causes alterations in cortical and hippocampal development. CB1 receptor knockout mice exhibit reduced levels of cortical progenitor cell proliferation, whereas FAAH-deficient mice show an increase in progenitor cell proliferation [40–42]. Subsequent observations in CB1 receptor knockout mice revealed abnormal cortical development, characterized by defective proliferation of progenitor cell subtypes occupying the ventricular zone (VZ) and SVZ, defective radial migration of immature cortical pyramidal neurons, deficits in axon pathfinding and aberrant subcortical projections [21].

At the level of eCB-mediated cellular signaling pathways, work from Bromberg and colleagues [43] show that CB1 receptor activation can affect over 20 transcription factors that, in turn, mediate neural precursor maintenance, neuronal differentiation and maturation. Specifically, CB1 receptor signaling activates downstream phosphatidylinositol 3-kinase (PI3K) and extracellular signal-related protein kinase (ERK) intracellular pathways to ultimately control transcriptional regulators such as CREB, STAT-3, PAX6 and β-catenin, which are all involved in the control of neural precursor cell proliferation and differentiation. In addition, CB1 receptor coupling with G_i-_mediated inhibition of adenylyl cyclase decreases cyclic adenosine monophosphate (cAMP), thus disinhibiting the ERK pathway via protein kinase A (PKA) [44,45]. Alternatively, CB1 receptor activation may involve downstream mammalian target of rapamycin complex 1 (mTORC1), a serine/threonine protein kinase that regulates cell growth, proliferation and survival [7]. However, the role of CB1 receptors in mTORC1 signaling during brain development remains to be fully elucidated. In the case of differentiating neurons, CB1 receptor activity has been shown to be coupled to the modulation of the neurogenic transcription factors COUP-TF II (Ctip2) and special AT-rich sequence-binding protein 2 (Satb2). While the mechanism through which this occurs has yet to be elucidated, CB1 receptors are positively coupled to Ctip2 and negatively to Satb2-mediated repression of Ctip2 [46,47]. As a result, CB1 receptor activity can fine-tune the transcriptional program through which neuronal differentiation occurs, where inactivation of CB1 receptors shifts differentiation in favor of upper-layer pyramidal neuron fate versus lower-layer pyramidal neuron fate. This change in neuronal fate-determination, in turn, affects motor function in adulthood [47].

## eCB Signaling in the Mature CNS

CB1 receptors are abundantly expressed throughout the central nervous system, notably in the basal ganglia, hippocampus, cerebral cortex, and cerebellum. Cortical and hippocampal CB1 receptor expression predominates in cholecystokinin-positive (CCK^+^) interneurons, but is also present at functional levels in glutamatergic terminals [48–51]. In the dorsal and ventral striatum, CB1 receptor expression is highly enriched in medium spiny neurons, particularly on direct pathway axons as they enter the globus pallidus en route to the substantia nigra [52–55].

eCBs are potent regulators of synaptic function and profoundly impact a wide range of neural functions, including cognition, motor control, feeding behaviors and pain perception. The primary mechanism through which eCBs regulate synaptic function is via retrograde signaling [56]. This is supported by evidence of presynaptic localization of CB1 receptors and their ability to inhibit neurotransmitter release, coupled with the postsynaptic localization of eCB synthesizing enzymes. Additionally, postsynaptic activity (increases in intracellular calcium and/or activation of G_q/11-_ linked GPCRs) increases eCB production. The first reports of retrograde eCB signaling emerged from groups studying forms of short-term plasticity known as depolarization-induced suppression of inhibition (DSI) [57–59] and depolarization-induced suppression of excitation (DSE) [59–66]. Subsequently, eCBs were also implicated in mediating forms of presynaptic long-term depression (eCB-LTD) at excitatory [67,68] and inhibitory terminals [69–72]. Recently, work from our group has characterized the role of BDNF-trkB receptor signaling in mobilizing eCB synthesis and release. In neocortical layer 2/3, acute application of BDNF rapidly suppresses GABAergic transmission via release of eCBs from the postsynaptic pyramidal cell, which in turn act in a retrograde manner to suppress presynaptic transmitter release [73]. This effect of BDNF is trkB-dependent, requires downstream PLC signaling and is independent of mGluR activation [71]. A follow-up study sought to examine the physiological relevance of BDNF-eCB interactions in the context of activity-dependent long-term depression at inhibitory synapses (iLTD). It was shown that theta burst stimulation (TBS) in layer 2/3 of mouse somatosensory cortical slices induces a form of eCB-mediated iLTD that is independent of mGluR activation but requires endogenous BDNF-trkB receptor signaling. Furthermore, the expression of this form of eCB-dependent iLTD requires activation of DAGL. Taken together, these results suggest that TBS can induce the release of endogenous BDNF, which triggers DAGL-dependent 2-AG mobilization and CB1 receptor-dependent iLTD [72]. BDNF also induces eCB release at cortical glutamatergic synapses [49]. These studies are a part of a growing body of evidence that BDNF and eCBs interact to regulate synaptic plasticity and further examination may provide clues to underlying pathologies of neurological and psychiatric disorders and suggest strategies for novel therapeutic targets and interventions.

## eCB Signaling in Autism Spectrum Disorders

A number of lines of evidence suggest that altered eCB signaling may underlie some of the behavioral impairments in ASD. For example, it has been shown that eCBs play a role in controlling emotional responses [14], behavioral reactivity to context [15] and social interaction [16]. Here, we provide an overview of the current evidence of changes to eCB signaling in experimental models of autism, specifically pertaining to the neuregulin 3 (Nlgn3) and Fragile X (FXS) mouse models. Collectively, these studies shed light on the link between alterations in eCB signaling in mediating ASD-related phenotypes and offer an alternative approach to therapeutic intervention and treatment.

Autism is neurodevelopmental in nature and atypical development of circuit connectivity has been suggested to underlie its key phenotypic features. Studies examining idiopathic forms of autism have revealed a set of genes involved in neurodevelopmental processes that mediate the formation, stabilization and pruning of synapses that consistently associate with autism-related phenotypes in animal models [74–76]. Specifically, several members of the neuroligin (NLGN) family of genes, postsynaptic cell adhesion molecules that interact with presynaptic neurexins (NRXNs) to control synapse development and function, have been identified in mouse models of ASDs [77]. Animal models have shown that NLGNs enhance synapse formation *in vitro* [78], but are not required for synapse formation *in vivo* [79]. In addition, NLGNs and NRXNs are important organizing molecules for excitatory glutamatergic and inhibitory GABAergic synapses in the mammalian brain [80,81]. Studies that suggest altered neurotransmission resulting from mutations or full gene deletion of NLGNs, particularly NLGN3, have prompted the investigation of the role of eCB signaling in mediating neurotransmitter release in the hippocampus and cortex [82–85].

A recent study by Foldy and colleagues [83] carefully examined one member of the NLGN family, neuroligin-3 (*NLGN3*) and its association with ASDs. Specifically, two distinct mutations in *NLGN3* have been linked with autism-related phenotypes in mouse, (1) a point mutation resulting in an R451C substitution in the neurexin binding domain (Nlgn3^R451C^) [86] and (2) a deletion of the *NLGN3* gene (Nlgn3 KO) [87]. Foldy and colleagues employ previously characterized mouse models of Nlgn3^R451C^ knock-in (KI) and Nlgn3 KO [84,85,88], which have both demonstrated autism-related phenotypes. However, each distinct *NLGN3* mouse model exhibits different effects in the context of synaptic and network function, where the Nlgn3^R451C^ KI results in a gain-of-function phenotype that is unrelated to the loss-of-function in the Nlgn3 KO mouse [82,85,89]. In this particular study, the authors examined GABAergic transmission in the hippocampus of both Nlgn3^R451C^ KI and Nlgn3 KO mice between inhibitory basket cells and pyramidal neurons, which are known to play a critical role in cognitive functions specific to the hippocampus [90]. Foldy and colleagues [83] found that CCK-expressing (CCK^+^) inhibitory basket cells in both Nlgn3^R451C^ KI and Nlgn3 KO mice exhibited an increase in inhibitory postsynaptic currents (IPSCs), most likely due to an increase in presynaptic release probability. The increase in GABA release probability results from deficits in tonic eCB signaling at presynaptic terminals of CCK^+^ interneurons [91]. The authors concluded that in the mouse hippocampus, Nlgn3 is required to establish the release machinery for tonic eCB release to CB1 receptor-containing synapses. Despite these promising results in exploring a particular mutation in the Nlgn3 gene and full gene deletion in mouse hippocampus, there remains the challenge of predicting the relevance of a given mutation for ASD-related phenotypes without directly assessing its effects in a synapse-specific and circuitry-specific manner.

Several studies exploring the Nlgn3^R451C^ mutation in the overlying cerebral cortex have revealed differential roles of *NLGN3* in regulating synaptic transmission. In an effort to add clarity to the regulatory role of *NLGN3*, Speed and colleagues [84] expanded on work from the Sudhof group [85] by further investigating the observed increase in inhibitory GABAergic transmission in layers II/III of the somatosensory cortex due to Nlgn3^R451C^ KI. In short, Speed and colleagues replicated experiments probing for altered GABAergic transmission in the cortex and narrowed the target mechanisms to altered eCB signaling, not an increase in interneuron number or inhibitory synaptic connections onto pyramidal neurons [84,85]. However, questions remain regarding the respective contribution of tonic eCb signaling and phasic eCB signaling to the altered GABAergic transmission in this model.

In two separate studies, dysfunctional eCB signaling was also identified in the FMR1 KO mouse, a model of FXS [92,93]. However, these studies appear to contradict one another in their conclusions, as Busquets-Garcia and colleagues [92] report gain of function, while Jung and colleagues [93] attribute loss of function of CB1 receptor activity in the absence of FMR1. On the one hand, Busquets-Garcia and colleagues [92] probed for changes in eCB signaling as a result of mGluR5 activation [94] in the CA1 region of the hippocampus in FMR1 mouse model of FXS and reported that CB1 receptor blockade in male FMR1 KO mice normalized cognitive impairment, nociceptive desensitization, susceptibility to audiogenic seizures, overactivation mTOR signaling and altered spine morphology. In addition, blockade of the CB2 receptor normalized anxiolytic-like behavior [92]. On the other hand, Jung and colleagues [93] examined a unique form of mGluR5-dependent long-term depression (LTD) at excitatory synapses of the ventral striatum and prefrontal cortex, which is mediated by the eCB 2-AG. This form of plasticity was absent in FMR1 KO mice, but could be restored with pharmacological enhancement of 2-AG signaling and thus corrected behavioral abnormalities in FMR1 KO mice [93]. Taken together, it is evident that the eCB signalosome is involved in mediating synaptic plasticity in multiple regions of the autistic brain. Further, it appears eCB signaling may exhibit regional specificity. Therefore, while the aforementioned studies in Nlgn3 and FMR1 mouse models of ASD serve to establish the importance of eCB signaling in mediating ASD-related behaviors, much remains to be elucidated by way of determining both up- and downstream mechanisms that are disrupted in ASDs.

## eCB Signaling in Clinical Studies

A deficit in theory of mind and empathy has been suggested to underlie atypical social behavior in individuals diagnosed with an ASD. Recent studies have revealed that deficits in reward system circuitry may underpin the lack of social motivation observed in children with ASDs [95–99]. Adaptive reinforcement of social interactions requires long-term synaptic plasticity at excitatory synapses of the nucleus accumbens (NAc) and is dependent on oxytocin (OT). OT, a neuropeptide produced by the paraventricular nucleus of the hypothalamus and released by the posterior pituitary, regulates prosocial behavior and its dysregulation has been implicated in social impairments [100,101]. A recent study by Wei and colleagues [102,103] demonstrated an obligatory role of anandamide in neural circuits of socialization in socially isolated rodents. This study suggested that OT acts as a social reinforcement signal to induce CB1 receptor-dependent long-term depression in medium spiny neurons. Therefore, the enhancement of OT to improve social deficits may be sufficient to trigger anandamide mobilization for proper functioning of the neural circuit. However, this hypothesis may apply to neurological disorders other than ASDs, such as schizophrenia, anxiety or personality disorders [104,105].

Clinical studies examining eye gaze tracking, electroencephalography and functional magnetic resonance imaging suggest clear differences in processing of social rewards in patients with ASDs compared to control subjects [106]. Atypical responses to social rewards from an early age may result in deficits in learning and interpreting the social world, which, in turn, can lead to social behavioral impairments in adulthood. On the other hand, human neuroimaging studies measuring striatal responses to social rewards (happy faces *vs*. neutral faces) found that common single nucleotide polymorphisms in *CNR1*, the gene coding for the human CB1 receptor, are associated with activity in the central striatal cluster in response to happy, but not disgusted, faces [107]. Given the central role of the ventral striatum in reward processing, this study suggests that changes in *CNR1* are linked to differences in the perception of, and sensitivity to, social rewards. A separate study of the same *CNR1* polymorphism investigated its effect on eye gaze in response to happy faces as opposed to disgusted facial expressions and found that eye gaze duration increased in individuals that carried the *CNR1* polymorphisms. However, there was no difference in eye gaze duration in response to faces expressing disgust [108]. In summary, current evidence strongly implicates alterations to the eCB system in animal models of ASDs and human patients that account for two phenotypic features of ASDs; (1) social reward perception and processing and (2) cortical development. Together, this body of work suggests that distinct elements of the eCB signaling system could be targets for novel therapeutics for treating and managing ASDs. While the ultimate goal is to uncover novel and effective targets, any potential therapeutic approach is unlikely to involve simply activating or inhibiting the eCB system to address specific behaviors or cognitive deficits related to ASDs. Instead, drug targets will require fine-tuning to the precise developmental timeline and to specific pathogenetic mechanisms underlying an individual patient’s disorder.

## Looking Forward and Taking the Next Steps

Elucidating the underlying mechanisms of aberrant developmental processes that lead to cognitive and behavioral deficits in ASDs is of paramount importance. It is becoming increasingly apparent from examining mature synapses and circuits that eCB signaling is altered in several neurological diseases. Further, considerable evidence suggests that eCB signaling plays an important role in controlling emotional responses, behavioral reactivity to context and social interaction, all of which are affected in ASDs. Therefore, it is plausible that eCB signaling should be viewed as a potential target for the development of novel therapeutics and treatment strategies in managing symptoms of ASDs in patients. However, there are several issues that must be addressed from the perspective of the etiology of ASDs rooted in abnormal cortical development.

First, we must clarify the aberrant developmental processes that result in deficits in synaptic modulation and circuit connectivity in the mature cortex that in turn underlie cognitive and behavioral symptoms of ASDs. Alternatively, eCB signaling may also play a role in the development of subcortical structures such as the hippocampus, nucleus accumbens and striatum, all of which have featured prominently in behavioral deficits associated with ASDs [83,103,109,110].

Second, the contribution of eCB signaling, be it loss of function or gain of function, during abnormal cortical development remains unresolved. Shedding light on the mechanism(s) through which eCB signaling is altered in development may provide insight as to whether symptoms of ASDs are a result of the developmental role of eCBs or their synaptic modulation, or a combination of the two. It may well be the case that the mechanisms of eCB signaling at the cellular/molecular level during neurodevelopment may differ from their synaptic arrangements in mature systems. ASDs are a uniquely human neuropathological condition. As a result, the few validated rodent models of ASDs remain limited in accurately recapitulating behavioral and cognitive impairments in human patients. To compensate for the short-comings of animal models, studies utilizing induced pluripotent stem cell (iPSC)- derived neurons have provided valuable insight into the pathogenesis of neurological disorders such as ASDs [111]. iPSC technology allows for the generation of patient-specific neurons, which have been used to model FXS [112], Rett Syndrome (RTT) [113], Angelman Syndrome [114], Chromosome 15q11.2–13.1 duplication syndrome (Dup15q) [115] and idiopathic autism [116] because the neuronal subtypes generated model neurons found in the developing brain. However, the eCB system has yet to be examined in the context of these aforementioned neurological disorders using iPSC-derived neurons. Indeed, iPSC-derived neurons may serve as an important model in determining the distinction between developmental eCB signaling mechanisms versus synaptic roles of eCBs.

Finally, these results should be followed up with a thorough examination of the aspects of the ASD phenotype that are a direct result of dysregulated eCB signaling. Advances in our understanding of eCB actions will undoubtedly facilitate pharmacological interventions and further, provide patients the best quality of life possible.
